# PReS-FINAL-2295: Correlations of health related quality of life reports completed by children with lupus and parents

**DOI:** 10.1186/1546-0096-11-S2-P285

**Published:** 2013-12-05

**Authors:** LN Moorthy, E Roy, MG Peterson, AL Hassett, R Cuttica, C Magalhaes, J Sato, S Appenzeller, R Marini, C Len, M Vasco, S Oliveira, M Rodrigues, R Almeida, F Sztajnbok, L Campos, A Jesus, C Silva, E Faugier, L Cobian, A Quintero-Del-Rio, A Adams, L Barinstein, E Chalom, K Onel, J Lopez-Benitez, L Ray, K Haines, P Hashkes, V Cartwright, D Kingsbury, N Singer, I Tomanova-Soltys, S Hong, A Reiff, T Lehman

**Affiliations:** 1 Pediatrics, Robert Wood Johnson Medical School-Univ of Medicine and Dentistry of NJ, New Brunswick, NJ, USA; 2 Hosp. for Special Surgery, New York, NY, USA; 3 Anesthiology, Univ. of Michigan, Ann Arbor, MI, United States; 4 Hosp. Pedro de Elizalde, Buenos Aires, Argentina; 5 Pediatrics, Univ. Estadual Paulista (UNESP), Sao Paulo, Brazil; 6 Faculty of Medical Science, State Univ. of Campinas, Campinas, Brazil; 7 Pediatrics, Faculty of Medical Science, State Univ. of Campinas, Campinas, Brazil; 8 Pediatrics, Universidade Federal de Sao Paulo (UNIFESP)/Escola Paulista de Medicina (EPM), Sao Paulo, Brazil; 9 Pediatrics, Univ. Federal do Rio de Janeiro, Instituto de Puericultura e Pediatria Martagao Gesteira, University City, Rio de Janeiro, Brazil; 10 Pediatrics, Univ. do Estado do Rio de Janeiro, Rio de Janeiro, Brazil; 11 Pediatrics, Children's Institute, Faculdade de Med da Univ. de Sao Paulo, Sao Paulo, Brazil; 12 Hosp. Infantil de México Federico Gómez, Mexico City, Mexico; 13 San Jorge Children's Hosp., Santurce, Puerto Rico; 14 Mount Sinai Hosp, New York City, NY, USA; 15 St. Barnabas Medical Center, Livingston, NJ, USA; 16 Univ of Chicago, Chicago, IL, USA; 17 Centro Medico La Costa, Asuncion, Paraguay; 18 Univ. of Mississippi Med Center, Jackson, MS, USA; 19 HUMC, Hackensack, NJ, USA; 20 Cleveland Clinic Foundation, Cleveland, OH, USA; 21 Legacy Emanuel Children's Hospital, Portland, OR, USA; 22 Metro Health Case Western Reserve Univ., Cleveland, OH, USA; 23 Winthrop University Hosp., Woodmere, NY, USA; 24 Univ. of Iowa, Iowa City, IA, USA; 25 Pediatrics, Children's Hospital Los Angeles, Los Angeles, CA, USA

## Introduction

Children with chronic diseases and parents often perceive disease impact differently. We previously found moderate correlations between child-parent reports of Simple Measure of Impact of Lupus Erythematosus in Youngsters (SMILEY).

## Objectives

To examine the correlation between child and parent health-related quality of life (HRQOL) scores in an expanded sample from the United States (US) and Latin America (LA).

## Methods

A cross-sectional multicenter cohort of children (≤ 18 years) with systemic lupus erythematosus (SLE) and parents completed the specific translation of SMILEY and Pediatric Quality of Life Inventory (PedsQL) Generic scales. Higher scores indicate better HRQOL for both scales. Independent and paired samples t-test were used to compare scores. We examined Spearman's correlation (rho) and intra-class correlation (ICC) between child and parent scores.

## Results

Mean child- (LA 67 ± 15, n = 123; US 64 ± 14, n = 162) and parent-report (LA 64 ± 16, n = 129; US 62 ± 15, n = 148) total SMILEY scores were higher for LA subjects. Some SMILEY domain scores (p < 0.05 for Effect of Self-child-report; and Social-child- and parent-reports); and child-report PedsQL total scores were also higher for LA subjects. US and LA child-report scores of SMILEY and PedsQL were higher (p < 0.05) compared to corresponding parent-report scores. For child-parent pairs, correlatons ranged from 0.3-0.7 (table 1), with lowest correlation in the Social domain.

**Table 1:** Child-parent correlations from US and LA (p values for rho and ICC <0

Scale and domains	US-rho (n); ICC	LA- rho (n); ICC
**SMILEY total**	0.5 (145); 0.5	0.5 (122); 0.5

Effect on self Limitations Social Burden of SLE	0.6 (144); 0.6 0.5 (145); 0.5 0.4 (145); 0.4 0.5 (144); 0.5	0.5 (122); 0.5 0.6 (122); 0.6 0.3 (122); 0.3 0.4 (121); 0.5

**Peds QL Generic total**	0.7 (138); 0.6	0.7 (100); 0.6

## Conclusion

As shown previously, children and parents may have varying attitudes about the impact of SLE on HRQOL. Because parents tend to overestimate disease impact, it remains critical to use both parent and child reports while assessing HRQOL.

## Disclosure of interest

L. Moorthy Grant/Research Support from: Arthritis Foundation Investigator Award supported this study, E. Roy: None declared, M. Peterson: None declared, A. Hassett Grant/Research Support from: Bristol-Myers Squibb (2013) Pfizer (2012) (not related to current study), R. Cuttica: None declared, C. Magalhaes: None declared, J. Sato: None declared, S. Appenzeller Grant/Research Support from: Fundacao de Amparo de Pesquisa do Estado de Sao Paulo (FAPESP) 2008/02917-0, Conselho Nacional de Desenvolvimento de Pesquisa CNPQ (471343/2011-0, 302205/2012-8)(not related to current study), R. Marini: None declared, C. Len: None declared, M. Vasco: None declared, S. Oliveira Grant/Research Support from: Roche and Novartis (not related to current study), M. Rodrigues: None declared, R. Almeida: None declared, F. Sztajnbok: None declared, L. Campos: None declared, A. Jesus: None declared, C. Silva: None declared, E. Faugier: None declared, L. Cobian: None declared, A. Quintero-Del-Rio: None declared, A. Adams Grant/Research Support from: Spoke once for Abbot in 2009 (not related to the current study), L. Barinstein: None declared, E. Chalom: None declared, K. Onel: None declared, J. Lopez-Benitez: None declared, L. Ray: None declared, K. Haines: None declared, P. Hashkes: None declared, V. Cartwright: None declared, D. Kingsbury: None declared, N. Singer: None declared, I. Tomanova-Soltys: None declared, S. Hong: None declared, A. Reiff: None declared, T. Lehman: None declared.

